# Electrospun PCL Fiber Mats Incorporating Multi-Targeted B and Co Co-Doped Bioactive Glass Nanoparticles for Angiogenesis

**DOI:** 10.3390/ma13184010

**Published:** 2020-09-10

**Authors:** Si Chen, Dagmar Galusková, Hana Kaňková, Kai Zheng, Martin Michálek, Liliana Liverani, Dušan Galusek, Aldo R. Boccaccini

**Affiliations:** 1 Centre for Functional and Surface Functionalized Glass, TnU AD, 911 01 Trenčín, Slovakia; dagmar.galuskova@tnuni.sk (D.G.); hana.kankova@tnuni.sk (H.K.); martin.michalek@tnuni.sk (M.M.); dusan.galusek@tnuni.sk (D.G.); 2 Institute of Biomaterials, University of Erlangen-Nuremberg, 91058 Erlangen, Germany; kai.zheng@fau.de (K.Z.); liliana.liverani@fau.de (L.L.); 3 Joint Glass Centre of the IIC SAS, TnU AD and FChFT STU, Studentska 2, 911 50 Trenčín, Slovakia

**Keywords:** electrospinning, bioactive glasses, sol-gel method, angiogenesis, tissue regeneration

## Abstract

Vascularization is necessary in tissue engineering to keep adequate blood supply in order to maintain the survival and growth of new tissue. The synergy of biologically active ions with multi-target activity may lead to superior angiogenesis promotion in comparison to single-target approaches but it has been rarely investigated. In this study, polycaprolactone (PCL) fiber mats embedded with B and Co co-doped bioactive glass nanoparticles (BCo.BGNs) were fabricated as a tissue regeneration scaffold designed for promoting angiogenesis. BCo.NBGs were successfully prepared with well-defined spherical shape using a sol-gel method. The PCL fiber mats embedding co-doped bioactive glass nanoparticles were fabricated by electrospinning using benign solvents. The Young’s moduli of the nanoparticle containing PCL fiber mats were similar to those of the neat fiber mats and suitable for scaffolds utilized in soft tissue repair approaches. The mats also showed non-cytotoxicity to ST-2 cells. PCL fiber mats containing BCo.BGNs with a relatively high content of B and Co promoted the secretion of vascular endothelial growth factor to a greater extent than PCL fiber mats with a relatively low B and Co contents, which demonstrates the potential of dual ion release (B and Co) from bioactive glasses to enhance angiogenesis in soft tissue engineering.

## 1. Introduction

Tissue engineering strategies, including the regeneration, repair and healing of bone [1], myocardium [2], skin [3] and other soft tissues, use temporary synthetic scaffolds to aid the natural healing of tissue defects. Electrospinning is a well-known method for fabricating micro- and nano-fiber mats from polymer solutions or melts [4]. This technique has been widely used in the manufacturing of tissue-engineered scaffolds and drug delivery systems [5]. Fiber mats constructed by electrospinning possess porosity and mechanical properties appropriate to applications in tissue engineering [5,6,7,8], being suitable as temporary scaffolds to support new tissue growth.

Various polymers can be processed by electrospinning to obtain fiber mats. For example, poly(epsilon-caprolactone) (PCL), as a common polymer used for the fabrication of tissue engineering scaffolds by electrospinning [9,10], possesses a low degradation rate in aqueous solutions and the degradation products are non-toxic. However, most solvents and mixtures of solvents and co-solvents suitable for electrospinning of PCL are toxic, e.g., chloroform/methanol, methylene chloride/methanol, methylene chloride/N,N-dimethylformamide (DMF), methylene chloride/toluene or tetrahydrofuran/DMF [11,12,13,14]. Due to drawbacks in the use of toxic solvents including the possible presence of traces of toxic solvents in the obtained fibers, the environmental impact and the laboratory workers safety, the concept of “green electrospinning” is receiving increasing attention [15]. Less toxic or non-toxic solvents (benign solvents, as defined in Reference [16]) have been screened out for electrospinning [17,18]. Alternative and benign solvents such as acetic acid, formic acid and acetone have been utilized for the “green electrospinning” of PCL [17,19,20,21,22].

Electrospinning can also be used to fabricate fiber mats for drug delivery or to obtain composites with other materials with additional functionalities such as antibacterial properties [23], promotion of angiogenesis [24] and bioactivity [25]. In tissue regeneration, angiogenesis is necessary and important to keep adequate blood supply to maintain the survival and growth of new tissues [26]. The literature describes various attempts at promoting angiogenesis by loading biomolecules, such as vascular endothelial growth factor (VEGF) [27], fibroblast growth factor [28], dimethyloxalylglycine [29] and platelet-derived growth factor [30], into electrospun fibers. However, several weaknesses, such as limited cost-effectiveness, unstable chemical structure and difficult sterilization, restrict the application of such growth factors in combination with electrospun biomaterials. On the contrary, inorganic bioactive particles, as relatively stable, low-cost and easily sterilizable agents, can be embedded into electrospun fibers to promote angiogenesis by their ability to release biological active, angiogenic ions [31].

Boldbaatar et al. fabricated SiO_2_-CoO glass microspheres and reported the synergistic effect of Si and Co dual ion release on the promotion of angiogenesis [32]. In our previous study, multi-targeted B and Co co-doped bioactive glasses based on 45S5 bioactive glass prepared by the melt quenching method were investigated, which showed the synergistic effect of the two ions in increasing the secretion of VEGF from bone marrow-derived stromal cells (ST-2 cell line), indicating a potentially positive effect on angiogenesis [33]. Both B and Co have been investigated as therapeutic ions that induce angiogenesis when released from bioactive glasses [34,35,36]. In this study, bioactive glass nanoparticles (BGNs) were synthesized by the sol-gel method, which provides the advantage of controlling the morphology and size of the particles in the nano or submicrometric scale [37]. BGNs can be dispersed in the polymer solution for electrospinning to construct fibrous composite scaffolds in which BGNs serve as suitable carriers for the delivery of therapeutic ions to impart biological functions to the fibers [22,38,39]. The incorporation of sol-gel borate bioactive glass particles [40] and dual ion doped silicate BGNs [41] in PCL electrospun fibers using benign solvents has been already reported in the literature. Differently to the previous studies, in this work, B and Co co-doped BGNs were embedded into PCL dissolved in acetic acid to fabricate composite fiber mats with dual ion delivery for angiogenesis promotion during tissue regeneration. One of the possible applications of these composite fibers is related, but not limited to, soft tissue engineering, in particular in cases in which angiogenesis plays a pivotal role (i.e., wound healing).

## 2. Materials and Methods

### 2.1. Synthesis of B and Co Co-Doped Bioactive Glass Nanoparticles (BCo.BGNs)

The method used for synthesizing BCo.BGNs was based on the modified Stöber synthesis reported by Zheng et al. [42]. The designed compositions of BCo.BGNs and the used precursors are shown in Table 1. Tetraethyl orthosilicate (TEOS, (C_2_H_5_O)_4_Si, 98%), boric acid (H_3_BO_3_, 99.5%) and cobalt nitrate hexahydrate (Co(NO_3_)_2_·6H_2_O, 98%) (all from Sigma-Aldrich), and calcium nitrate tetrahydrate (Ca(NO_3_)_2_ 4H_2_O, 99.1%, VWR International GmbH) were used as reactants. First, solution A was prepared by mixing 10 mL of ammonium hydroxide solution (28%, Sigma-Aldrich), 8.12 mL of ethanol and 12.38 mL of deionized water. Solution B was prepared by dissolving 2.25 mL of TEOS in 25 mL of ethanol (96%, VWR International GmbH). Solution B was poured into solution A under vigorous stirring, and then boric acid was added immediately afterwards. After stirring for 30 min, calcium nitrate tetrahydrate and cobalt nitrate hexahydrate were added into the solution. After 2 h, the solution was centrifuged at 7830 rpm (Centrifuge 5430R, Eppendorf, Hamburg, Germany) for 15 min. The nanoparticles were collected and washed 3 times with deionized water and ethanol. The collected nanoparticles were dried at 60 °C in an oven overnight, then heated to 700 °C at a heating rate of 2 °C/min and calcined at this temperature for 3 h. Finally, it was cooled naturally in the oven. The nanoparticles with different compositions were labelled as B0Co0, B5Co2 and B10Co4 (Table 1).

### 2.2. Fabrication of PCL Fiber Mats Containing BCo.BGNs (PCL-BCo.BGNs Mats)

20% w/v PCL (80 kDa, Sigma Aldrich) was dissolved in glacial acetic acid (≥98%, VWR International GmbH) at room temperature and stirred overnight. The solution was then placed in an ultrasonic bath for 1 h. BCo.BGNs were dispersed in the PCL/acetic acid solution (15 wt % with respect to PCL) under stirring for 10 min. The mixture was immediately used to fabricate the fiber mats. Electrospinning was carried out using a commercially available setup (Starter Kit 40 KV Web, Linari Engineering srl, Valpiana (GR), Italy). The electrospinning parameters were based on a previous study reported by Liverani et al. [19], which included an applied voltage of 15 kV, a needle-target distance of 11 cm and a mixture flow rate of 0.4 mL/h. The prepared fiber mats were denoted as PCL, PCL-B0Co0, PCL-B5Co2 and PCL-B10Co4.

### 2.3. Materials Characterization

The morphology of BCo.BGNs and PCL-BCo.BGNs mats was examined by scanning electron microscopy (SEM) (Auriga Base, Zeiss, Jena, Germany). The chemical structure of the samples was characterized by Fourier transform infrared spectroscopy in attenuated total reflectance mode (ATR-FTIR) (Shimadzu IR Affinity-1S, Kyoto, Japan) with 42 spectral scans at a resolution of 4 cm^−1^ and with a wavenumber in the range 400–4000 cm^−1^. The Maximum – Minimum normalization was carried out for comparison of the peaks. The minimum value of the spectrum was transformed into a 0, and the maximum value was transformed into a 1. All the other values were transformed into the range between 0 and 1 by the formula: transformed data = (original value − minimum value)/(maximum value − minimum value). The element distribution of PCL-BCo.BGNs mats was detected by energy-dispersive X-ray spectroscopy (EDX) (OXFORD instruments, X-Max, 50 mm^2^, Abingdon, United Kingdom). The compositions of BCo.BGNs were measured by inductively coupled plasma-optical emission spectrometry (ICP-OES) (5100 SVDV, Agilent Technologies, Mulgrave, Australia), with 1.2 kW radio frequency power and 0.55 L/min carrier gas flow. BCo.BGNs were digested in the digestion solution, prepared by mixing 6 mL of HCl (30%, Merck), 2 mL HNO_3_ (67%–69%, Analytika Ltd.) and 1 mL HF (47%, VWR Chemicals), using a microwave digestion unit (SPEEDWAVE 4, Berghof, Eningen, Germany) in two heating steps: 180 °C (10 min) and 210 °C (40 min).

#### 2.3.1. Ion Release and Bioactivity of BCo.BGNs in SBF

The release of Si, P, Ca, B and Co ions from the BCo.BGNs was determined by ICP-OES. BCo.BGNs (10 mg each) were immersed in 15 mL of simulated body fluid (SBF) following the procedure described in literature [43] for 1, 3 and 7 days at 37 °C. The composition of 1000 mL SBF is: NaCl 8.035 g, NaHCO_3_ 0.355 g, KCl 0.225 g, K_2_HPO_4_·3H_2_O 0.231 g, MgCl_2_·6H_2_O 0.311 g, 1.0 M HCl 39 mL, CaCl_2_ 0.292 g, Na_2_SO_4_ 0.072 g, Tris 6.118 g, 1.0 M HCl 0–5 mL [43]. Afterwards, the solution was centrifuged, and the supernatant was collected and measured by ICP-OES. After immersion, the BCo.BGNs were washed with deionized water, dried in an oven at 60 °C and then measured by SEM and ATR-FTIR. The concentrations of ions released by the PCL-BCo.BGNs mats in culture medium after cell cultivation for 7 days were detected by inductively coupled plasma-mass spectrometry (ICP-MS) (7900, Agilent Technologies, Tokyo, Japan).

#### 2.3.2. Degradation of PCL-BCo.BGNs Mats in SBF

For this test, PCL-BCo.BGNs mats were fixed on plastic holders with 10 mm inner diameter, thus the samples could sink to the bottom of the flask containing SBF. In this way, samples were immersed in 15 mL of SBF for 1, 3 and 7 days at 37 °C. After immersion, all samples were washed with deionized water, dried at room temperature and examined by SEM and ATR-FTIR.

#### 2.3.3. Wettability

The water contact angle of PCL-BCo.BGNs mats was measured with a Drop Shape Analysis System (DSA30, Krüss GmbH, Hamburg, Germany) at room temperature. The associated DSA software (DSA4 2.0, Krüss GmbH, Hamburg, Germany) was used to process the data.

#### 2.3.4. Mechanical Tests

The mechanical properties of PCL-BCo.BGNs mats were measured by a standard uniaxial tensile test (5960 Dual Column Tabletop Testing System, Instron^®^, Darmstadt, Germany) at room temperature. The samples were cut into 20 mm × 4 mm rectangles and fixed in paper frames, according to Reference [22]. Samples were elongated at a speed of 10 mm/min with an initial length of 10 mm and using a 100 N load cell. Stress-strain curves were used to determine the Young’s modulus. Eight replicas were measured for each sample type.

### 2.4. Viability Test of ST-2 Cells

The PCL-BCo.BGNs mats were cut and mounted on scaffold holders (Scaffdex, Sigma Aldrich, St. Louis, MO, USA) with 10 mm inner diameter, thus the samples could sink to the bottom of the well containing culture medium. The samples were disinfected by exposure to ultra-violet (UV) light for 1 h on both sides. Drop seeding with a drop of 100 μL/sample containing 2.5 × 10^5^ cells/mL was performed on the surface of the mats in a 24-well plate and incubated at 37 °C in humidified atmosphere of 5% CO_2_ for 1 and 7 days. The culture medium after cultivation for 7 days was collected for detection of released ions by ICP-MS. ST-2 cells (Deutsche Sammlung von Mikroorganismen und Zellkulturen GmbH, Braunschweig, Germany), a bone marrow-derived stromal cell line, were used. The cell culture medium was Roswell Park Memorial Institute (RPMI) medium (Thermo Scientific, Schwerte, Germany) supplemented with 10% v/v fetal bovine serum (Sigma Aldrich^®^, Munich, Germany) and 1.0% v/v penicillin streptomycin (Thermo Scientific, Schwerte, Germany). The cytotoxicity was measured using a WST-8 assay (CCK-8, Sigma-Aldrich). 1% v/v WST-8/medium solution was added on the surface of the mats in a 24-well plate. The WST-8/medium solution was also incubated without cells and used as a control (blank). The plates were incubated for 3 h. Then, 100 μL of WST-8/medium solutions from each well in the 24-well plate were transferred to a 96-well plate. The absorbance of the solutions was detected with a microplate reader (PHOmo, anthos Mikrosysteme GmbH, Germany) at 450 nm.

### 2.5. Fluorescent Staining

Cellular actin cytoskeleton was stained with 4 µL/mL of rhodamine phalloidin (Thermo Scientific, Schwerte, Germany). Cell nuclei were stained with 1 µL/mL of 4’,6-diamidino-2-phenylindole (DAPI) (Thermo Scientific, Schwerte, Germany). The fluorescent images of the cells were obtained by a fluorescence microscope (Axio Scope A1, Carl-Zeiss, Jena, Germany).

### 2.6. VEGF Measurement

The secretion of VEGF from ST-2 cells was detected by a Mouse VEGF Enzyme-Linked Immunosorbent Assay (ELISA) kit (RayBiotech, Peachtree Corners, GA, USA). The medium of the ST-2 cells cultivated on the PCL-BCo.BGNs mats was used to carry out this assay. This procedure followed the instructions supplied by the manufacturer.

### 2.7. Statistical Analysis

The statistical analysis was performed by one-way analysis of variance (ANOVA) using Statistical Package for the Social Sciences (SPSS) 11.5 (Chicago, IL, USA). Probability (P) values P < 0.05 were considered to indicate statistically significant differences. The results were expressed as mean ± standard deviation (SD).

## 3. Results

**Figure 1:**
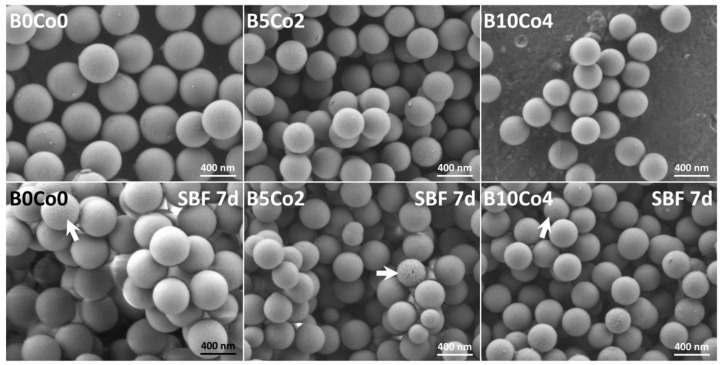
Scanning electron microscopy (SEM) images of the BCo.BGNs before and after immersion in simulated body fluid (SBF) for 7 days. The white arrows indicate nanoparticles exhibiting corrosion pits.

Figure 1 shows SEM images of the BCo.BGNs. The particles are seen to be monodispersed with a narrow size distribution and exhibit a well-defined spherical shape. No aggregates or precipitates are observed. The nanoparticles without B and Co doping showed the largest size (457 ± 20 nm in diameter). The sizes of B5Co2 and B10Co4 nanoparticles were similar (361 ± 9 nm and 331 ± 21 nm in diameter). After immersion in SBF for 7 days, a few corrosion pits, which are indicated by the white arrows on the micrographs, but no hydroxycarbonate apatite (HCA) crystals, were observed on the surface of the BCo.BGNs.

Table 2 shows the compositions of the BCo.BGNs measured by ICP-OES. B and Co were successfully doped into the bioactive glass nanoparticles using the sol-gel method. However, it should be noted that the actual compositions differ significantly from the projected (nominal) compositions of BCo.BGNs listed in Table 1. Such a gap between the nominal and actual compositions has been reported previously in similar ion-doped particles synthesized by the sol-gel method [42,44,45]. Nevertheless, in terms of the actual B and Co contents, the concentrations change consistently with the projected nominal compositions.

**Figure 2:**
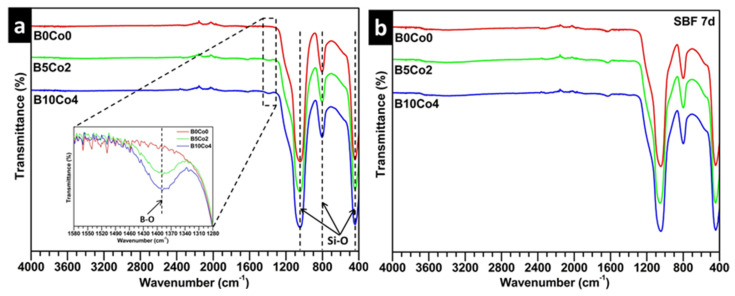
Fourier transform infrared (FTIR) spectra of the BCo.BGNs before (**a**) and after (**b**) immersion in SBF for 7 days. Main bands are indicated and discussed in the text.

Figure 2a shows the FTIR spectra of the BCo.BGNs. The peaks at 443 and 803 cm^−1^ and the broad-band in the 1100–900 cm^−1^ range are attributed to the bending and stretching vibrations of Si–O–Si bonds [46,47]. The peak at 1388 cm^−1^ represents the B–O stretching vibration of trigonal (BO_3_) [48]. The relative intensity of this peak increased with the increase of B content. The spectra of the BCo.BGNs after immersion in SBF for 7 days (Figure 2b) do not show any difference compared with those of the BCo.BGNs before immersion in SBF. The peaks related to phosphate cannot be identified in the spectra. The result is consistent with SEM observations after a 7-day immersion period in SBF (Figure 1).

**Figure 3:**
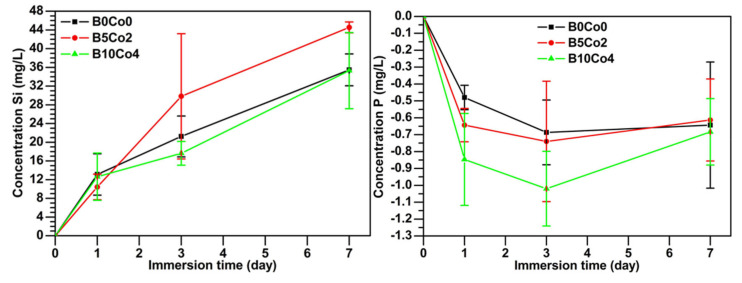

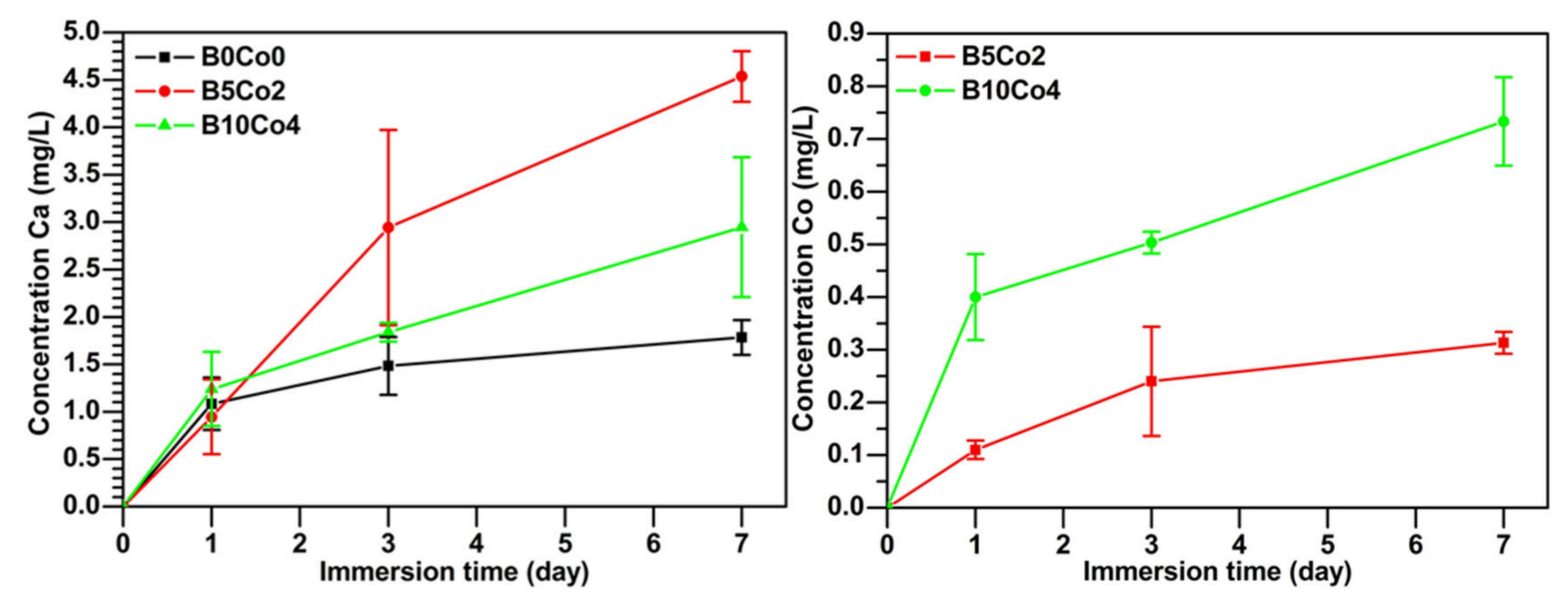
The cumulative ion (Si, P, Ca, Co) release concentration of BCo.BGNs after immersion in SBF for 7 days, measured with ICP-OES.

The ion release kinetics for BCo.BGNs in SBF is shown in Figure 3. The cumulative concentration of all elements, except P, increases with immersion time, which indicates that BCo.BGNs gradually degraded in SBF, as expected. The small negative cumulative concentration of P may indicate the formation of a small amount of phosphate in the SBF, induced by nanoparticles, which precipitates out of the solution as HCA. Combined with the results of SEM and FTIR, the data suggests that the bioactivity of the nanoparticles is not strong enough to form visible and detectable HCA crystals. This is not anticipated to be an issue in the context of the intended applications of the present biomaterials (wound dressings), where mineralization effects are not required. The cumulative concentrations of Co were low. In addition, due to the different Co content of the BCo.BGNs, Co was released in different concentrations. The concentration of B released from B10Co4 and B5Co2 was very low, close to the detection limit of the instrument. The trends are therefore not shown due to their low statistical significance.

**Figure 4:**
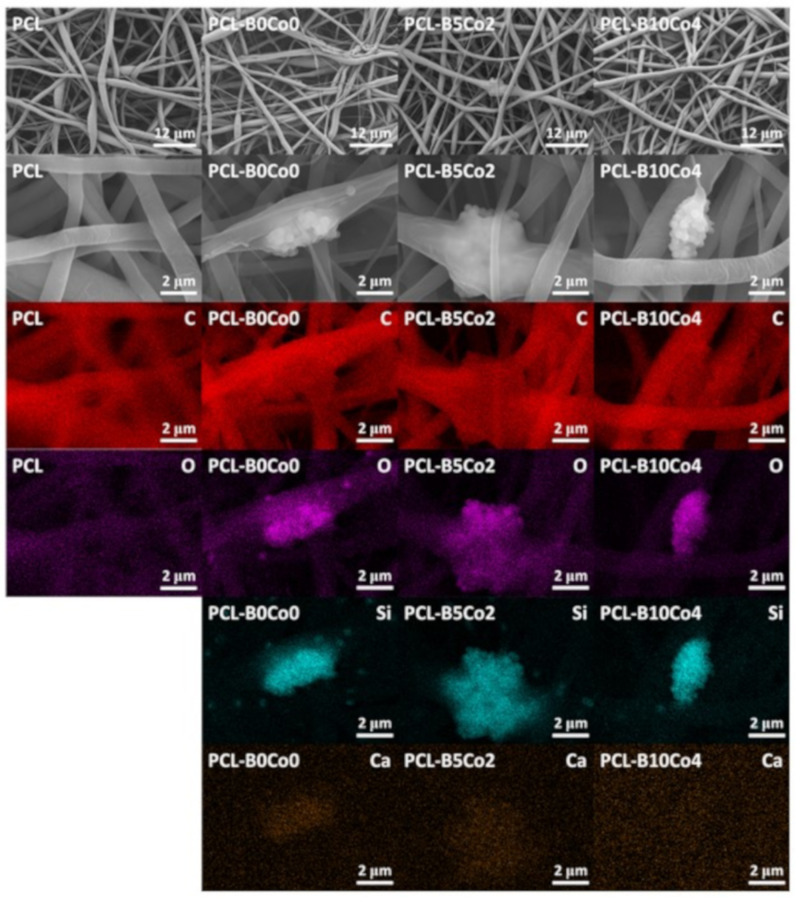
SEM micrographs and energy-dispersive X-ray spectroscopy (EDX) element maps of PCL-BCo.BGNs mats.

Figure 4 shows SEM images and EDX element maps of the PCL-BCo.BGNs mats. All samples exhibit fibrillary morphology without beads. Only a few fibers in the PCL-B0Co0 sample were seen to fuse together. In Table 3, the average fiber diameter is shown, indicating that PCL-B0Co0 mats had smaller diameter than the other samples. The standard deviation and minimum/maximum fiber diameter show a wide distribution of fiber diameters, which can also be observed in Figure 4. The Young’s modulus of PCL-B0Co0 was lower than the one of the other samples. In the EDX analysis (Figure 4), the carbon map clearly indicates the PCL component in the composite fibers, while the silicon map shows the aggregated and dispersed nanoparticles. Oxygen is present in both PCL and bioactive glasses, showing the profiles of both the fibers and nanoparticles. Enrichment of calcium is observed in the region where the nanoparticles were aggregated, except in PCL-B10Co4, which is consistent with the significantly lower calcium content in this sample in comparison to PCL-B0Co0 and PCL-B5Co2 mats, as reported in Table 2. The last column of Table 3 shows the water contact angle results indicating the wettability of PCL-BCo.BGNs mats. The contact angles of all samples, which are around 134°–143°, did not show significant differences and confirm a hydrophobic behavior. It should be mentioned that even if some agglomeration of particles was detected, a surface modification of nanoparticles (that could possibly improve their distribution in the solution), was not considered, as such particle surface modification could introduce toxic remnants in the fibers.

The study of PCL-BCo.BGNs mats by FTIR (Supplementary Figure S1) revealed the C–H stretching vibration peaks (2943 and 2864 cm^−1^) of –CH_2_, the asymmetric and symmetric C–O–C stretching vibration peaks (1240 and 1165 cm^−1^) of ester groups and the C=O stretching vibration peak (1722 cm^−1^), all attributed to PCL [22]. The relevant peaks of BCo.BGNs cannot be observed due to the prevalent amount of PCL.

**Figure 5:**
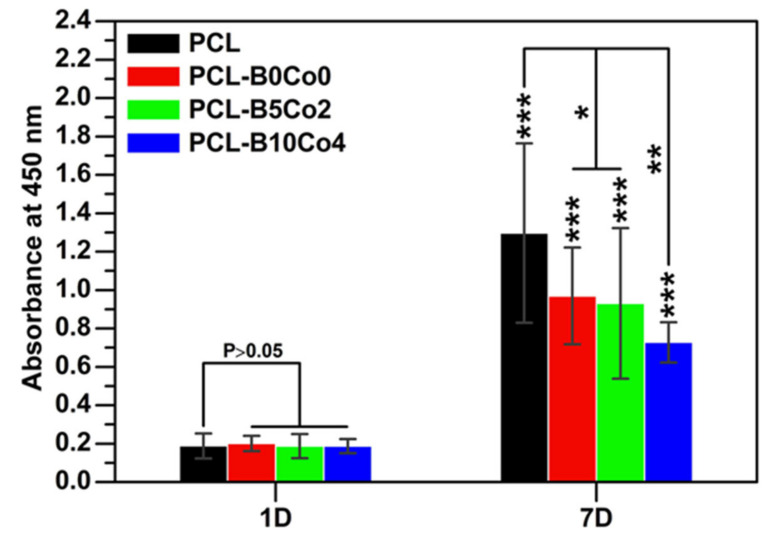
WST-8 results from cell culture experiments with ST-2 cells directly cultured on PCL-BCo.BGNs mats for 1 and 7 days (0.01 < *P < 0.05, 0.001 < **P < 0.01, ***P < 0.001, mean ± SD, N = 9).

**Figure 6:**
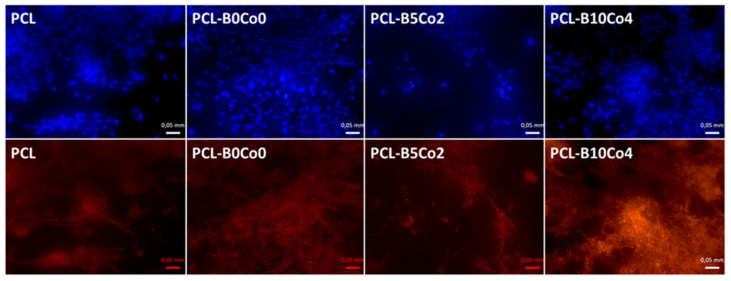
DAPI (blue) and rhodamine (red) fluorescent images of ST-2 cells directly cultured on PCL-BCo.BGNs mats for 7 days.

Cell viability (WST-8) results are shown in Figure 5. The data does not show any significant difference in the viability of cells cultivated on the PCL-BCo.BGNs mats at 1 day. After 7 days, the viability of all samples was significantly higher than after 1 day. The cells cultivated on PCL fiber mats possessed the highest viability, while cells cultivated on PCL-B10Co4 mats exhibited the lowest viability. Figure 6 shows the DAPI and rhodamine phalloidin fluorescent images of ST-2 cells 7 days after seeding. DAPI and rhodamine stained cell nuclei and cytoskeleton with blue and red fluorescence, respectively. The fluorescent images indicate that the ST-2 cells indeed infiltrated into the PCL-BCo.BGNs mats.

**Figure 7:**
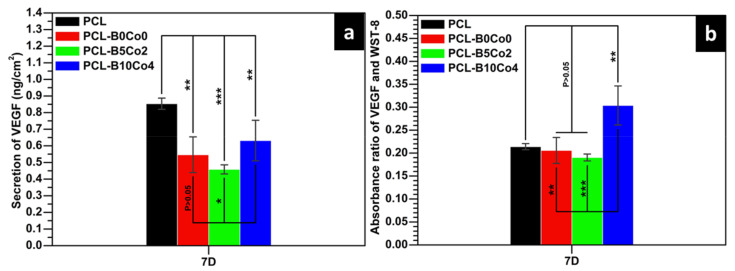
(**a**) Vascular endothelial growth factor (VEGF) secretion from ST2 cells directly cultured on PCL-BCo.BGNs mats for 7 days, (**b**) absorbance ratio of VEGF and WST-8 (0.01 < *P < 0.05, 0.001 < **P < 0.01, ***P < 0.001, mean ± SD, N = 3).

The total VEGF secretion from ST-2 cells cultivated on the fiber mats after 7 days is shown in Figure 7a. Cells cultivated on neat PCL showed the highest value of total VEGF secretion. The VEGF secretion of cells seeded on PCL-B10Co4 was higher than that on PCL-B0Co0 and PCL-B5Co2. To properly evaluate the results obtained from total VEGF secretion, it is possible to analyze the absorbance ratio of VEGF and WST-8, which reflects the secretion of VEGF per unit number of cells [33], and it is shown in Figure 7b. The PCL-B10Co4 sample showed the highest secretion of VEGF per unit number of cells. In addition, the concentrations of Si, B and Co released by PCL-BCo.BGNs mats after 7–day cultivation are shown in Table 4. The concentrations of ions released from PCL-BCo.BGNs mats are seen to be lower than those of neat BCo.BGNs. B could not be detected in the culture medium of PCL-B5Co2, which is consistent with the B release behavior of BCo.BGNs. However, both B and Co were detected in the culture medium of PCL-B10Co4. These results indicate that PCL-BCo.BGNs mats with a high content of B and Co may exhibit a synergistic effect of the two ions, leading to the increase of VEGF secretion from ST-2 cells after 1 week of cell culture.

## 4. Discussion

There are reports in the literature on the fabrication of B-doped bioactive glass nanoparticles [49,50], but, to the best of our knowledge, there are very limited data available on the fabrication and characterization of Co-doped B-containing bioactive glass nanoparticles. Rad et al. reported that the content of B in their B-doped bioactive glass nanoparticles, prepared using an acid-base catalyzed sol-gel method, was quite similar to their designed compositions [49]. However, the prepared nanoparticles were aggregated and their shape often deviated from spherical shape [49]. Under acidic conditions, silica particles that grow more slowly than under basic conditions tend to aggregate into a three-dimensional (3D) network structure [51]. Because of the increased probability of reactant collisions, the slow growth rate and the 3D network structure are beneficial for the reaction between TEOS and boric acid. In this study, nanoparticles were fabricated using a basic catalyzed sol-gel method, resulting in well-defined spherical shape nanoparticles (Figure 1). However, the contents of B and Co were lower than the designed compositions. This gap between the actual and the nominal compositions has been reported in previous studies on sol-gel-derived bioactive glass nanoparticles [42,43,44,45,52]. It is caused not only by the different growth rate and different 3D structure of the particles, due to different catalysis systems, but also by the different reaction balances and time points at which the precursors are added [42].

In the study reported by Zheng et al. [42], bioactive glass nanoparticles usually show significant bioactivity with obvious HCA crystals grown on the particles upon immersion in SBF. For BCo.BGNs prepared in this work, the results of SEM and FTIR did not show any HCA crystals or phosphate or carbonate peaks (Supplementary Figure S2). Especially, the small negative cumulative concentration of P may indicate the precipitation of a small amount of HCA during immersion in SBF. The low bioactivity may be due to the lower content of Ca compared to nanoparticles synthesized in previous studies [42]. Furthermore, the cumulative concentration of B was very low in the leaching solution. However, chemical analysis by ICP-OES confirmed the presence of B in BCo.BGNs. Since boric acid was added together with TEOS at the beginning of the process, we assume that B was probably concentrated in the center of the nanoparticle and therefore could not be released during the first 7 days of immersion.

After electrospinning, the average fiber diameter of PCL-B0Co0 mats was lower than in other samples and a few fibers were blended. The electrospinning experimental record confirmed that during the fabrication of PCL-B0Co0 mats, the humidity was higher (29%–30%) than during the electrospinning of the other compositions (23%–24%). Vrieze et al. [53] reported the effect of humidity on electrospinning. They suggested that an increase in humidity would result in the increase or decrease of fiber diameter depending on the type of polymer used. Vrieze et al. concluded that this effect was related to the solvent evaporation rate [53]. The lower solvent evaporation rate under high humidity might also be able to explain the fiber blending. All PCL-BCo-BGNs mats showed wide distributions of fiber diameters, which has also been reported in the literature from studies using benign solvents for electrospinning of PCL [19,22,40,52]. A single acetic acid solvent system causes a wide distribution of fiber diameters. The addition of formic acid to the solvent system improves the homogeneity of the distribution of fiber diameters [19,22,54]. Van der Schueren et al. proposed that acetic acid, with low conductivity, results in inhomogeneous charge distribution in the solvent and causes a broadening of the distribution of fiber diameters [19]. In this study, formic acid was not used in the solvent system due to the possible rapid degradation of the highly reactive nanoparticles.

The mechanical properties of electrospun fiber mats are very important for their use as scaffold for tissue regeneration. According to the study by Hollister et al., an adequate range of Young’s modulus to support deformation in soft tissue scaffolds pliability should be in the 0.4–350 MPa range [55]. The Young’s moduli of all PCL-BCo.BGNs mats matched this range, which shows that, from the mechanical point of view, these mats could be applied as soft tissue regeneration scaffolds. In addition, the Young’s modulus of PCL-B0Co0 mats was lower than that of the other samples, this trend being consistent with the average fiber diameter. Some studies reporting on the effect of humidity during electrospinning on the mechanical properties of fiber mats have concluded that the mechanical properties of fiber mats prepared at high humidity were lower due to the insufficient bonding between fibers in the mat caused by a solid PCL surface layer which formed by phase separation [56]. Such phase separation results by the cooling of the electrospun fiber caused by solvent evaporation [56]. The work of Pelipenko et al. revealed that a broad range of nanofiber diameters could be prepared through humidity control during electrospinning, which could further affect the mechanical properties, such as Young’s modulus [57]. However, the work of Vrieze et al. suggests that an increase in humidity might result either in the increase or decrease of the fiber diameter, depending on the type of polymer used [53]. Although some studies indicate that there may be a correlation between high humidity during the fabrication of PCL fibers and their low Young’s modulus, the problem of the humidity effect on the mechanical properties of electrospun fiber mats is complex, depending on both the solvent system and the polymer used.

The WST-8 results on ST-2 cells cultivated on PCL-BCo.BGNs mats showed that cells on all samples significantly proliferated from day 1 to day 7, which suggests that all fiber mats were cytocompatible. The viability of the ST-2 cells cultured on PCL fiber mats for 7 days was significantly higher than that of the cells cultured on the mats containing nanoparticles. This is probably due to the high local pH of the microenvironment attributed to the presence of calcium oxide at the surface of bioactive glass nanoparticles in direct cell tests. Other studies published on the topic reported the same phenomenon: Quinlan et al. reported that the viability of human umbilical vein endothelial cells cultured on a collagen glycosaminoglycan scaffold was higher than that of cells cultured on the same scaffolds but containing bioactive glasses [35], and Noh et al. reported that the viability of preosteoblast cells (MC3T3-E1) cultured in a culture dish was higher than that of cells cultured on poly(lactic acid) nanofibers containing bioactive glasses [58].

Vascularization is vital during tissue regeneration [26] and VEGF is an essential cytokine during angiogenesis [59], one of the vascularization mechanisms [60]. The results of the ELISA test showed that, similar to our previous research results [33], the sample with a high content of B and Co (PCL-B10Co4) significantly promoted the secretion of VEGF in comparison to samples with either no or low B and Co content (PCL-B0Co0 and PCL-B5Co2). On the other hand, few studies have reported ion release data from electrospun fiber mats during cell culture. In the present case, the concentrations of B and Co, which are directly related to VEGF secretion from the ST-2 cells, were measured by ICP-MS. The secretion of VEGF was increased by B and Co co-doped bioactive glass nanoparticles under relatively low cumulative concentrations of B (~70 μg/L) and Co (~32 μg/L), which indicates a synergistic effect of the two ions in the present multi-targeted B and Co co-doped bioactive glass nanoparticles on the promotion of VEGF secretion. Clearly, the combination of these two angiogenic ions enables the reduction of the required concentration of each of them to allow angiogenesis, which avoids potential cytotoxic effects due to an excessive concentration.

## 5. Conclusions

In this study, B and Co co-doped bioactive glass nanoparticles with a well-defined spherical shape were successfully prepared using the sol-gel method. Furthermore, electrospun PCL fiber mats containing the multi-targeted B and Co co-doped bioactive glass nanoparticles were successfully fabricated. The cell viability test showed that composite PCL-BCo.BGNs fiber mats were biocompatible to ST-2 cells. Most importantly, the fiber mats containing B- and Co-doped nanoparticles could promote VEGF secretion with a low release concentration of B and Co in comparison with the high release concentration of B or Co reported in the literature for bioactive glasses doped with only one of these ions. All composite PCL electrospun mats exhibited a clear fibrous shape without beads. The Young’s modulus of all composite fiber mats is in the adequate range to enable the pliability of scaffolds for soft tissue engineering. In summary, the PCL fiber mats embedding multi-targeted B and Co co-doped bioactive glass nanoparticles demonstrated their potential as scaffolds for soft tissue engineering applications in which promotion of angiogenesis is required.

## Figures and Tables

**Table 1:** Designed compositions of BCo.BGNs in mol% and reagents used.

Samples	B_2_O_3_ (H_3_BO_3_)	CaO (Ca(NO_3_)_2_·4H_2_O)	CoO (Co(NO_3_)_2_·6H_2_O)	SiO_2_ ((C_2_H_5_O)_4_Si)
**B0Co0**	0	20	0	80
**B5Co2**	5	20	2	73
**B10Co4**	10	20	4	66

**Table 2:** Actual compositions of BCo.BGNs in mol% measured with inductively coupled plasma-optical emission spectrometry (ICP-OES).

Samples	B	Ca	Co	Si
**B0Co0**	0	4.44 ± 0.02	0	96 ± 2
**B5Co2**	0.61 ± 0.04	4.28 ± 0.09	0.106 ± 0.008	95 ± 2
**B10Co4**	4.03 ± 0.04	1.7 ± 0.1	0.195 ± 0.008	94 ± 1

**Table 3:** Average fiber diameter (compared with PCL: 0.01 < *P < 0.05, 0.001 < **P < 0.01, ***P < 0.001, mean ± standard deviation (SD), N = 180), minimum and maximum fiber diameter and Young’s modulus (compared with PCL: 0.01 < *P < 0.05, 0.001 < **P < 0.01, ***P < 0.001, mean ± SD, N = 7), and water contact angle (mean ± SD, N = 5) of PCL-BCo.BGNs mats.

Samples	Average Fiber Diameter (nm)	Minimum Fiber Diameter (nm)	Maximum Fiber Diameter (nm)	Young’s Modulus (MPa)	Water Contact Angle (°)
**PCL**	916 ± 603	105	2912	10 ± 2	139 ± 3
**PCL-B0Co0**	770 ± 555 *	105	2932	7 ± 1 *	137 ± 1
**PCL-B5Co2**	924 ± 561 ^(P > 0.05)^	149	2640	7 ± 3 ^(P > 0.05)^	137.8 ± 0.8
**PCL-B10Co4**	1022 ± 484 ^(P > 0.05)^	158	2797	11 ± 3 ^(P > 0.05)^	137 ± 3

**Table 4:** The concentrations of Si, B and Co released by PCL-BCo.BGNs mats after a 7-day cell cultivation period, measured with inductively coupled plasma-mass spectrometry (ICP-MS) (mean ± SD, N = 9).

Samples	Si (mg/L)	B (μg/L)	Co (μg/L)
**PCL-B0Co0**	15 ± 3	0	0
**PCL-B5Co2**	6 ± 2	0	28 ± 4
**PCL-B10Co4**	4 ± 2	70 ± 11	32 ± 7

## Supplementary Materials

The following are available online at https://www.mdpi.com/1996-1944/13/18/4010/s1, Figure S1: The FTIR spectra of the PCL-BCo.BGNs mats. Main bands are indicated and discussed in the text, Figure S2: The SEM images of the PCL-BCo.BGNs mats after the immersion in SBF for 7 days.
