# Supplementary material for: Electrospun PCL Fiber Mats Incorporating Multi-Targeted B and Co Co-Doped Bioactive Glass Nanoparticles for Angiogenesis
Source: Materials (Basel). 2020 Sep 10;13(18):4010. doi: 10.3390/ma13184010 (PMC7557727; doi:10.3390/ma13184010)
Supplement: Supplementary file 1 [file materials-13-04010-s001.pdf]

materials

Supplementary Information

Electrospun PCL Fiber Mats Incorporating Multi-Targeted B and Co Co-Doped Bioactive Glass Nanoparticles for Angiogenesis

Si Chen [1,2,*], Dagmar Galusková [1], Hana Kaňková [1], Kai Zheng [2], Martin Michálek [1,2], Liliana Liverani [2], Dušan Galusek [1,3] and Aldo R. Boccaccini [2,*]

1 Centre for Functional and Surface Functionalized Glass, TnU AD, 911 01 Trenčín, Slovakia; dagmar.galuskova@tnuni.sk (D.G.); hana.kankova@tnuni.sk (H.K.); martin.michalek@tnuni.sk (M.M.); dusan.galusek@tnuni.sk (D.G.)
2 Institute of Biomaterials, University of Erlangen-Nuremberg, 91058 Erlangen, Germany; kai.zheng@fau.de (K.Z.); liliana.liverani@fau.de (L.L.)
3 Joint Glass Centre of the IIC SAS, TnU AD and FChFT STU, Studentska 2, 911 50 Trenčín, Slovakia
* Correspondence: si.chen@tnuni.sk (S.C.); aldo.boccaccini@ww.uni-erlangen.de (A.R.B.); Tel.: +49-9131-852-8601

Received: 26 July 2020; Accepted: 1 September 2020; Published: 10 September 2020

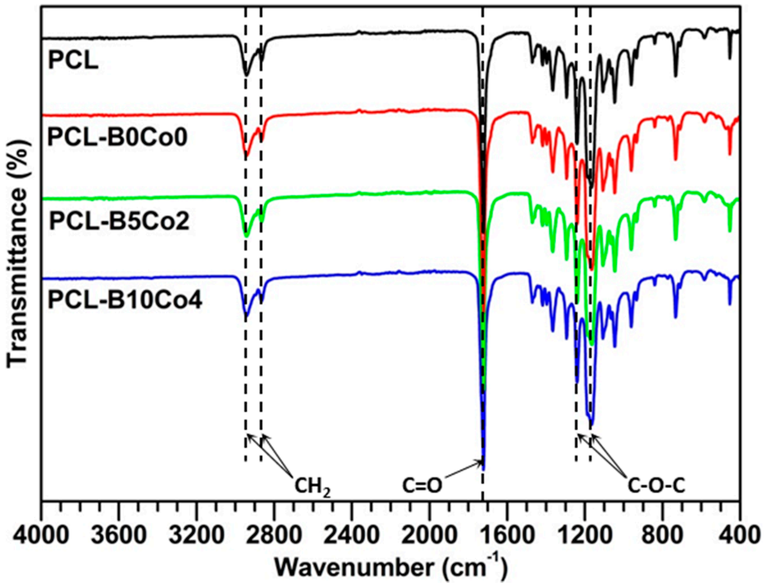

Figure S1. The FTIR spectra of the PCL-BCo.BGNs mats. Main bands are indicated and discussed in the text.

Figure S2 showed the SEM images of PCL-BCo.BGNs mats after immersed in SBF for 7 days. It did not show any significant difference compared with PCL-BCo.BGNs mats before immersed in SBF.

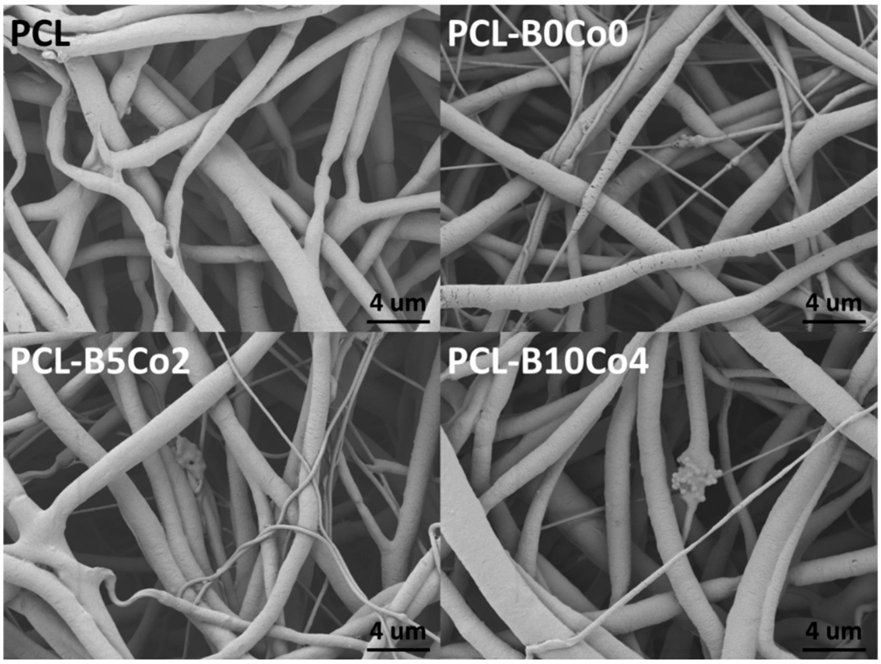

Figure S2. The SEM images of the PCL-BCo.BGNs mats after the immersion in SBF for 7 days.

© 2020 by the authors. Submitted for possible open access publication under the terms and conditions of the Creative Commons Attribution (CC BY) license (http://creativecommons.org/licenses/by/4.0/).
